# *C. elegans* Germline-Deficient Mutants Respond to Pathogen Infection Using Shared and Distinct Mechanisms

**DOI:** 10.1371/journal.pone.0011777

**Published:** 2010-07-26

**Authors:** Michael TeKippe, Alejandro Aballay

**Affiliations:** Department of Molecular Genetics and Microbiology, Duke University Medical Center, Durham, North Carolina, United States of America; Massachusetts General Hospital, United States of America

## Abstract

Reproduction extracts a cost in resources that organisms are then unable to utilize to deal with a multitude of environmental stressors. In the nematode *C. elegans*, development of the germline shortens the lifespan of the animal and increases its susceptibility to microbial pathogens. Prior studies have demonstrated germline-deficient nematodes to have increased resistance to Gram negative bacteria. We show that germline-deficient strains display increased resistance across a broad range of pathogens including Gram positive and Gram negative bacteria, and the fungal pathogen *Cryptococcus neoformans*. Furthermore, we show that the FOXO transcription factor DAF-16, which regulates longevity and immunity in *C. elegans*, appears to be crucial for maintaining longevity in both wild-type and germline-deficient backgrounds. Our studies indicate that germline-deficient mutants *glp-1* and *glp-4* respond to pathogen infection using common and different mechanisms that involve the activation of DAF-16.

## Introduction

Studies in a variety of species ranging from insects to mammals have demonstrated that reproduction extracts a cost in terms of lifespan [1], [2]. Additional studies have linked successful reproduction with reductions in immunocompetence [3], [4], [5], [6]. The connection between reproduction and longevity has perhaps been most thoroughly studied in the nematode *Caenorhabditis elegans* [7]. Lack of germline proliferation in *C. elegans*, either by laser ablation of the germ cell precursors or by mutation, causes an increase in lifespan [8], [9]. This lifespan extension is dependent on the FOXO transcription factor DAF-16 [8], [10], [11]. The activity of DAF-16 is tightly regulated by a wide variety of external stimuli and internal control mechanisms. The insulin/PI3K/Akt pathway has been shown to control longevity by regulating DAF-16 [12]. Interestingly, lack of *C. elegans* germline results in an extended lifespan that requires intestinal DAF-16 [10]. This lifespan extension observed in germline-ablated nematodes appears to only involve DAF-16 and otherwise be largely independent of the insulin/PI3K/Akt pathway [7].

In addition to its role in longevity, DAF-16 has also been shown to play a role in regulating *C. elegans* immunity. Nematodes that overexpress DAF-16 or have increased activation of DAF-16 through the mutation of *daf-2* exhibit enhanced resistance to a variety of pathogens [13], [14], [15], [16], [17]. In addition, several DAF-16 transcriptional targets appear to play key roles in antimicrobial defense [18]. Recent studies suggest that the germline may play a role in modulating DAF-16-mediated immune responses in the nematode [19], [20]. Epistasis analysis indicates that *daf-16* mutations completely or partially suppress the enhanced resistance to Gram negative pathogens *Pseudomonas aeruginosa* and *Serratia marcescens* of germline-deficient animals [19], [20], [21].

Here, we show that the germline-deficient mutant *glp-1*, which has a mutation in a Notch family receptor critical for germline development [22], [23], exhibits enhanced resistance to a wide array of microbes, including the Gram positive pathogen *Enterococcus faecalis*, the Gram negative pathogen *Salmonella enterica*, and the fungal pathogen *Cryptococcus neoformans*. Our studies show that *glp-4* mutants are more resistant to *E. faecalis*, *P. aeruginosa*, and *C. neoformans*, but unlike the germline-deficient mutant *glp-1*, *glp-4* mutants are not more resistant to *S. enterica* and exhibit wild-type lifespan when grown on live *E. coli*, the usual food of *C. elegans* in the laboratory. When grown on killed *E. coli*, the lifespan of *glp-4* mutants is significantly longer than that of wild-type animals, suggesting that *glp-4* animals may be hypersusceptible to certain microorganisms. Our studies suggest that germline-deficient mutants exhibit enhanced immune responses against microorganisms using common and different mechanisms.

## Results

### *glp-4* and *glp-1* mutants exhibit different responses to pathogen infections

**Figure 1:**
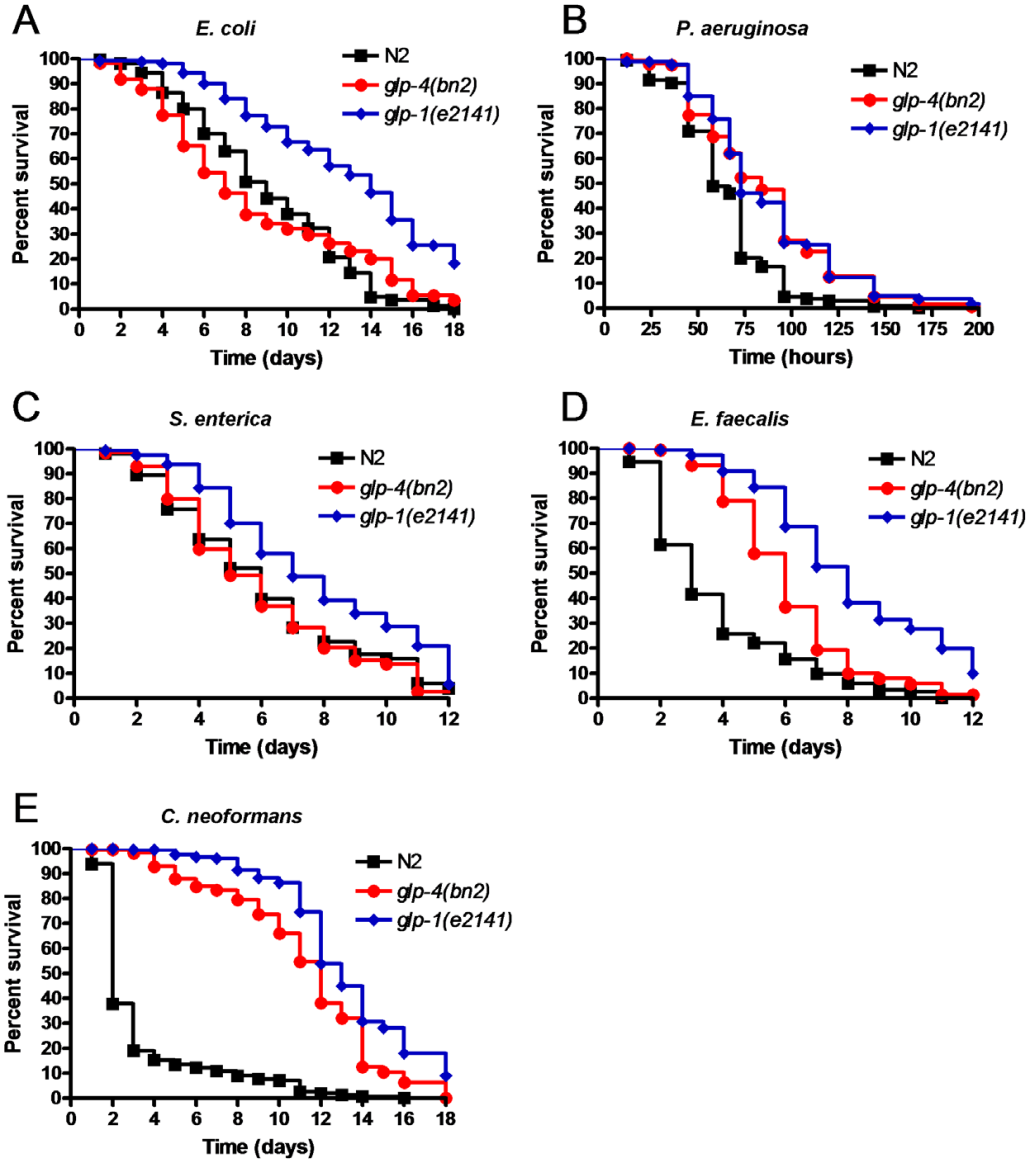
Germline-deficient mutants exhibit different responses to pathogens. Wild-type, *glp-4(bn2)* mutant, and *glp-1(e2141)* mutant nematodes were exposed to (**A**) *E. coli*, (**B**) *P. aeruginosa*, (**C**) *S. enterica*, (**D**) *E. faecalis*, and (**E**) *C. neoformans.* Significant differences were found when wild-type nematodes were compared to *glp-1(e2141)* mutants on all five pathogens (P<0.0001). Significant differences were also found when wild-type nematodes were compared to *glp-4(bn2)* mutants on *P. aeruginosa* (P<0.0001), *E. faecalis* (P<0.0001), and *C. neoformans* (P<0.0001) but not on *E. coli* (P = 0.9433) nor *S. enterica* (P = 0.1485). 160–300 nematodes were used for each condition.

We analyzed the survival of *glp-4* and *glp-1* mutant nematodes when infected with a wide array of pathogens, including the Gram negative bacteria *P. aeruginosa* and *S. enterica*, the Gram positive bacterium *E. faecalis*, and the fungal pathogen *C. neoformans*. *glp-1* mutants exhibit enhanced resistance to all these pathogens (Figure 1), which suggests that the loss of the germline leads to an increase in the general immune function of the nematode. As the increased longevity and pathogen resistance observed in *glp-1* mutants has been attributed to the absence of the germline and not a specific function of the *glp-1* gene [8], [20], one might expect other germline-deficient mutants to show a similar lifespan extension and pattern of broad-range resistance to pathogen infection. However, *glp-4* mutants failed to show enhanced resistance to *S. enterica* (Figure 1C) while exhibiting enhanced resistance to *P. aeruginosa*, *E. faecalis*, and *C. neoformans* (Figures 1B, 1D, and 1E). In addition, the longevity of *glp-4* mutants was comparable to that of N2 wild-type animals when grown on *E. coli* (Figure 1A).

**Figure 2:**
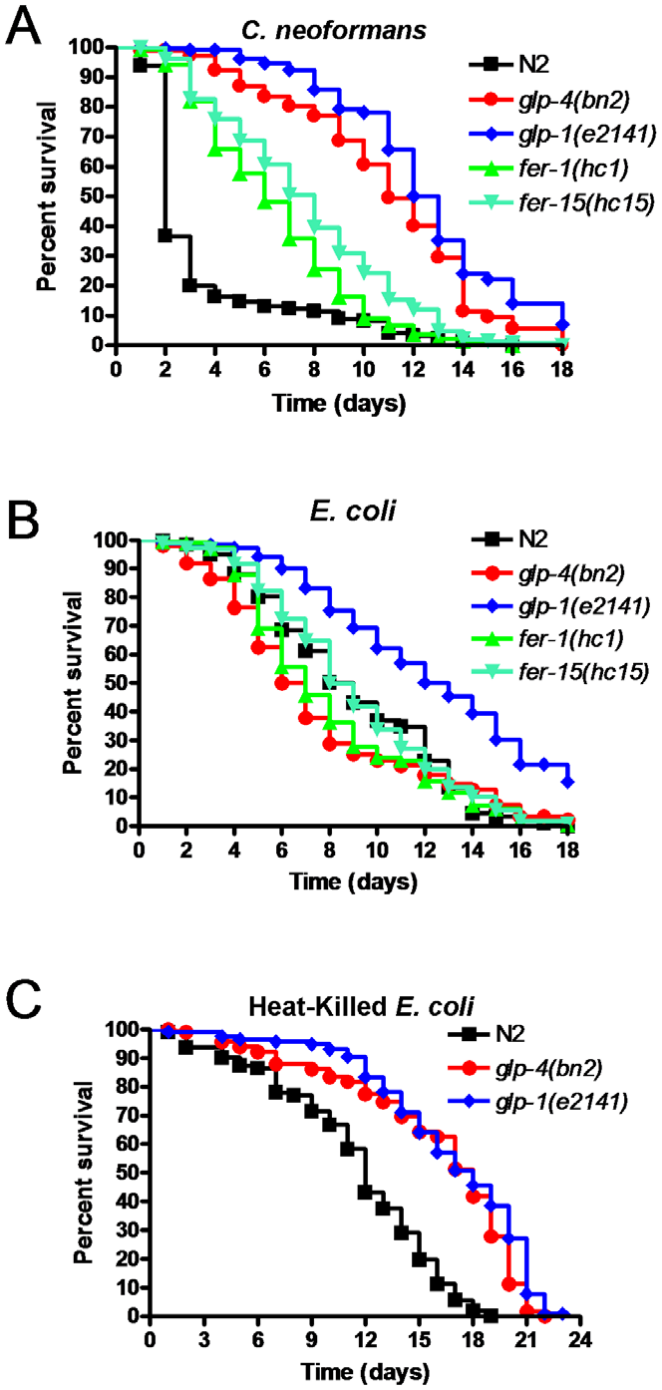
The increased resistance germline-deficient mutants *glp-1* and *glp-4* is independent of effects on matricide. (**A**) Wild-type, *glp-4(bn2)* mutant, *glp-1(e2141)* mutant, *fer-1(hc1)* mutant, and *fer-15(hc15)* mutant nematodes were exposed to *C. neoformans*. When compared to wild-type nematodes, all four mutants showed significant differences (P<0.0001). Significant differences were also found when *glp-4(bn2)* mutants or *glp-1(e2141)* mutants were compared to *fer-1(hc1)* (P<0.0001; P<0.0001 respectively) or to *fer-15(hc15)* (P<0.0001; P<0.0001 respectively) mutants. (**B**) Wild-type, *glp-4(bn2)* mutant, *glp-1(e2141)* mutant, *fer-1(hc1)* mutant, and *fer-15(hc15)* mutant nematodes were exposed to *E. coli*. When compared to wild-type nematodes, only *glp-1(e2141)* mutants showed significant increases in resistance (P<0.0001). (**C**) Wild-type, *glp-4(bn2)* mutant, and *glp-1(e2141)* mutant nematodes were placed on lawns of heat-killed *E. coli* and survival was measured. When compared to wild-type nematodes, both *glp-4(bn2)* mutants (P<0.0001) and *glp-1(e2141)* mutants (P<0.0001) showed significant differences. 120–300 nematodes were used in each condition.

It is possible that the enhanced resistance to pathogens observed in *glp-1* and *glp-4* mutants was simply due to lack of matricide. Matricide is a process in which eggs hatch inside the still living nematode, ultimately leading to the death of the adult animal. Often the rates of matricide are much higher in sicker nematodes, and consequently matricide can be a factor in survival assays. As *glp-4* and *glp-1* mutants lack a fully functional germline, they do not suffer any matricide. To test whether the increased resistance observed in *glp-4* and *glp-1* mutants was due to a lack of matricide, we compared the survival of N2 wild-type, *glp-4* mutant, and *glp-1* mutant nematodes to that of *fer-1* and *fer-15* mutant nematodes. Both *fer-1* and *fer-15* do not suffer from matricide due to the lack of fertilization since their sperm production is affected [24], [25]. As shown on Figure 2A, *fer-1* and *fer-15* mutants are more resistant to *C. neoformans*, a pathogen that induces very high rates of matricide. The survival of *fer-1* and *fer-15* mutants is similar to that of N2 wild-type animals when grown on *E. coli* (Figure 2B), indicating that their enhanced resistance to *C. neoformans* is a consequence of lack of matricide rather than enhanced overall longevity. However, since *fer-1* and *fer-15* mutants are not as resistant to *C. neoformans* infection as *glp-4* or *glp-1* mutants (Figure 2A), lack of matricide cannot account for the enhanced resistance of *glp-4* or *glp-1* mutants.

### *glp-4* mutant nematodes are susceptible to live *E. coli*

Consistent with the idea that the enhanced resistance to pathogen infection of germline-deficient mutants is not simply due to a lack of matricide, the increase in resistance to *P. aeruginosa* exhibited by *glp-1* mutants has been found to be due to increased intestinal DAF-16 activity [20]. Additionally, DAF-16 activation has been shown to be critical in promoting longevity [8], [9], [26]. Typically, these longevity studies have been performed by growing the nematodes on lawns of live *E. coli* [8], [9], [26]. By these standards, it appears that *glp-4* mutants do not exhibit an increase in longevity as they do not live longer on *E. coli* (Figure 1A).

To further study the lifespan of germline-deficient mutants, we performed survival assays using both *glp-4* and *glp-1* mutants grown on heat-killed *E. coli.* Under these conditions, we found that both *glp-4* and *glp-1* mutant nematodes live considerably longer than N2 wild-type animals (Figure 2C). This suggests that both *glp-1* and *glp-4* mutant strains have increased longevity and that *glp-4* mutants are susceptible to live *E. coli*, as they do not live proportionally as long on live *E. coli* as they do on heat-killed *E. coli*. This also provides additional support that the lifespan extension observed in *glp-1* and *glp-4* mutants on *C. neoformans* is not due merely to the lack of matricide as very little matricide occurs on heat-killed *E. coli*.

### DAF-16 activity is required for the enhanced longevity and resistance to *C. neoformans* of *glp-4* and *glp-1* mutants

**Figure 3:**
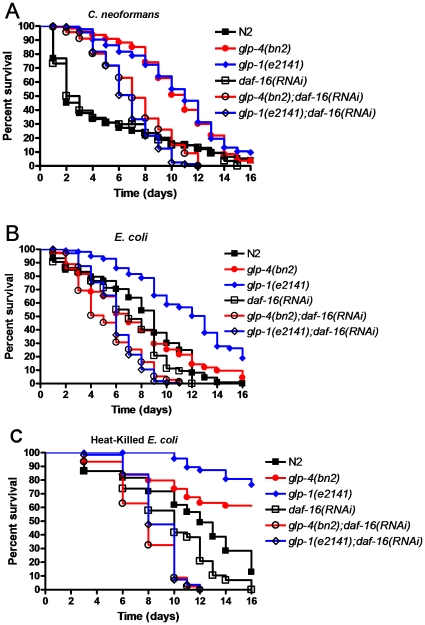
Increased resistance and longevity in germline-deficient mutants requires DAF-16. Wild-type, *glp-4(bn2)* mutant, and *glp-1(e2141)* mutant nematodes grown on *E. coli* carrying a vector control plasmid or expressing *daf-16* dsRNA were exposed to (**A**) *C. neoformans*, (**B**) *E. coli*, or (**C**) heat-killed *E. coli*. Significant differences were found when *glp-4(bn2);daf-16(RNAi)* worms were compared to vector control-treated *glp-4(bn2)* nematodes on *C. neoformans* (P<0.0001), *E. coli* (P<0.0001), and heat killed *E. coli* (P<0.0001). Likewise, significant differences were found when *glp-1(e2141);daf-16(RNAi)* nematodes were compared to vector control-treated *glp-1(e2141)* nematodes on *C. neoformans* (P<0.0001), *E. coli* (P<0.0001), and heat-killed *E. coli* (P<0.0001). When wild-type nematodes were compared to *daf-16(RNAi)* animals, significant differences were seen on *E. coli* (P<0.0237) and heat-killed *E. coli* (P = 0.0122) but not on *C. neoformans* (P = 0.7084). 60–300 nematodes were used for each condition.

To determine the involvement DAF-16 may have in the longevity of *glp-4* and *glp-1* mutants and their immune function against *C. neoformans*, we used RNAi to decrease DAF-16. Consistent with previous observations [15], *daf-16* RNAi has no effect on the resistance to *C. neoformans* of N2 wild-type animals (Figure 3A). However, *daf-16* RNAi inhibits the enhanced resistance to *C. neoformans* of both *glp-4* and *glp-1* mutants (Figure 3A). Inhibition of DAF-16 by RNAi also shortens the lifespan of *glp-4* and *glp-1* grown on lawns of both live and killed *E. coli* (Figures 3B and 3C), suggesting that DAF-16 function is required for the increased longevity of both *glp-1* and *glp-4* mutants.

**Figure 4:**
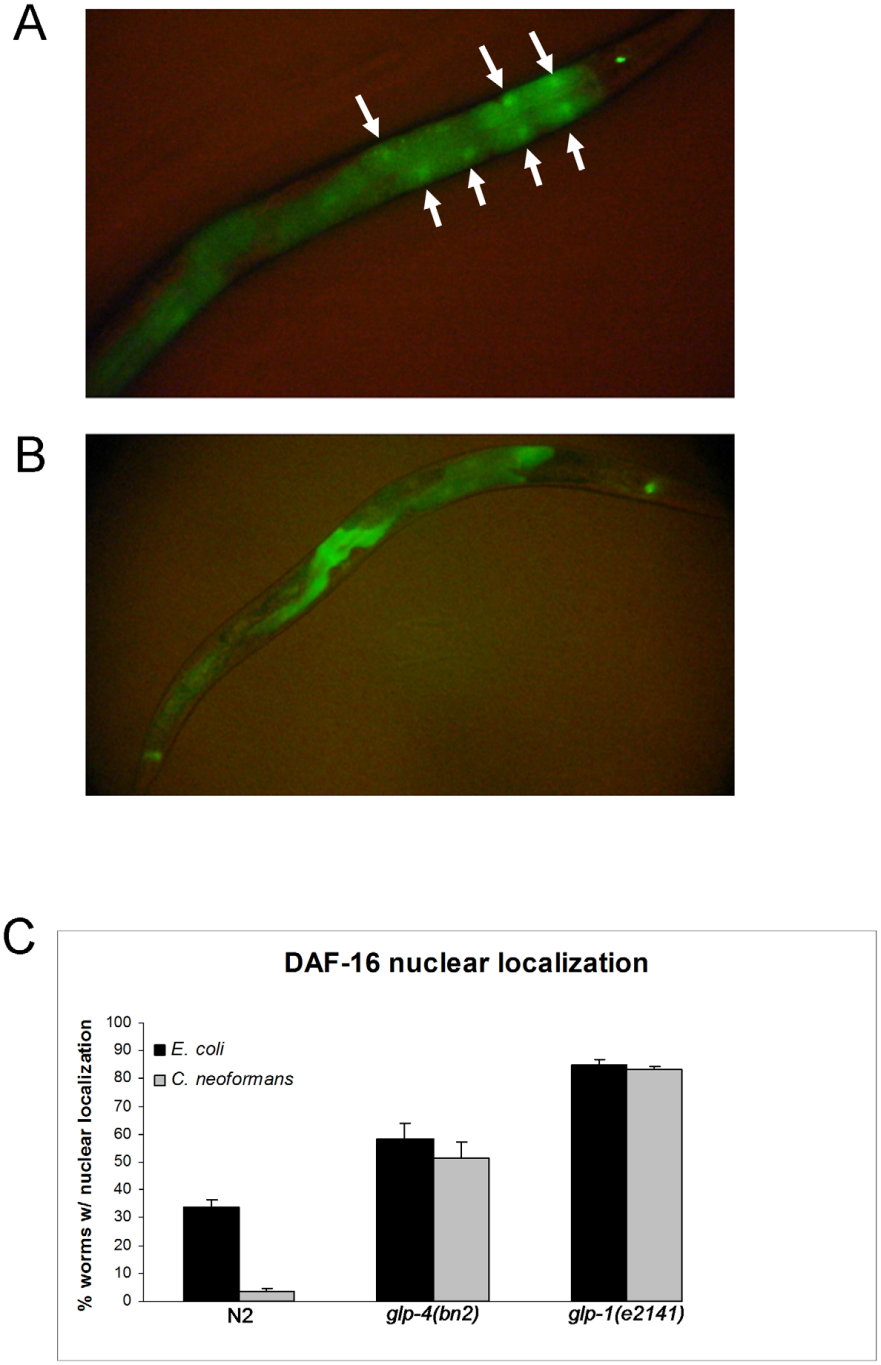
The germline-deficient mutants *glp-1* and *glp-4* have higher levels of DAF-16 activation than wild-type animals regardless of pathogen exposure. (**A**) A *glp-1(e2141)* mutant nematode expressing a *daf-16:gfp* transgene under control of *Pgly-19* after exposure to *E. coli*. (**B**) A wild-type nematode expressing a *daf-16:gfp* transgene under control of *Pgly-19* after exposure to *E. coli*. (**C**) Wild-type, *glp-4(bn2)* mutant, and *glp-1(e2141)* nematodes expressing transgenic DAF-16:GFP under control of *Pgly-19* were exposed to either *E. coli* or *C. neoformans* and categorized as predominately nuclear or cytoplasmic as described in Section 3.4.8. Significant differences were found when *glp-4(bn2)* mutants were compared to wild-type on both *E. coli* (P = 0.0003) and *C.neoformans* (P<0.0001). Likewise, significant differences were also found when *glp-1(e2141)* mutants were compared to wild-type on both *E. coli* (P<0.0001) and *C. neoformans* (P<0.0001). No significant differences were found when comparing the two *glp-4(bn2)* groups (P = 0.4363) nor with the two *glp-1(e2141)* groups (P = 0.7802), but there were significant differences in DAF-16 localization between the wild-type nematodes on *E. coli* and *C. neoformans* (P = 0.0002).

These results, together with the observation that DAF-16 RNAi does not seem to affect the immune function of the nematode unless the animals exhibit increased levels of DAF-16 activation [14], [15], suggest that *glp-1* and *glp-4* mutants may have high levels of DAF-16 activity. To measure the level of DAF-16 activation, we crossed both *glp-1* and *glp-4* mutant nematodes to a strain containing a transgene that creates a DAF-16::GFP fusion protein under the regulation of the intestinal-specific promoter of *gly-19* [27]. We chose to utilize a DAF-16::GFP fusion protein regulated by an intestinal promoter since DAF-16 is specifically activated in the intestinal cells of *glp-1* mutants and that activation is required for lifespan extension in *glp-1* mutants [10], [11], [26]. We then scored nematodes as having predominately nuclear localization of DAF-16 (Figure 4A) or as diffusely cytoplasmic (Figure 4B). We found that although fewer *glp-4* mutants showed DAF-16 intestinal activation than *glp-1* mutants, *glp-4* mutant animals were significantly more likely to exhibit DAF-16 activation in the intestinal cells than N2 wild-type animals regardless of whether they were exposed to *E. coli* or *C. neoformans* (Figure 4C). These results indicate that intestinal DAF-16 is activated in both germline-deficient mutants regardless of pathogen exposure.

### *glp-1* RNAi enhances longevity and pathogen resistance in wild-type animals but not in *glp-4* animals

**Figure 5:**
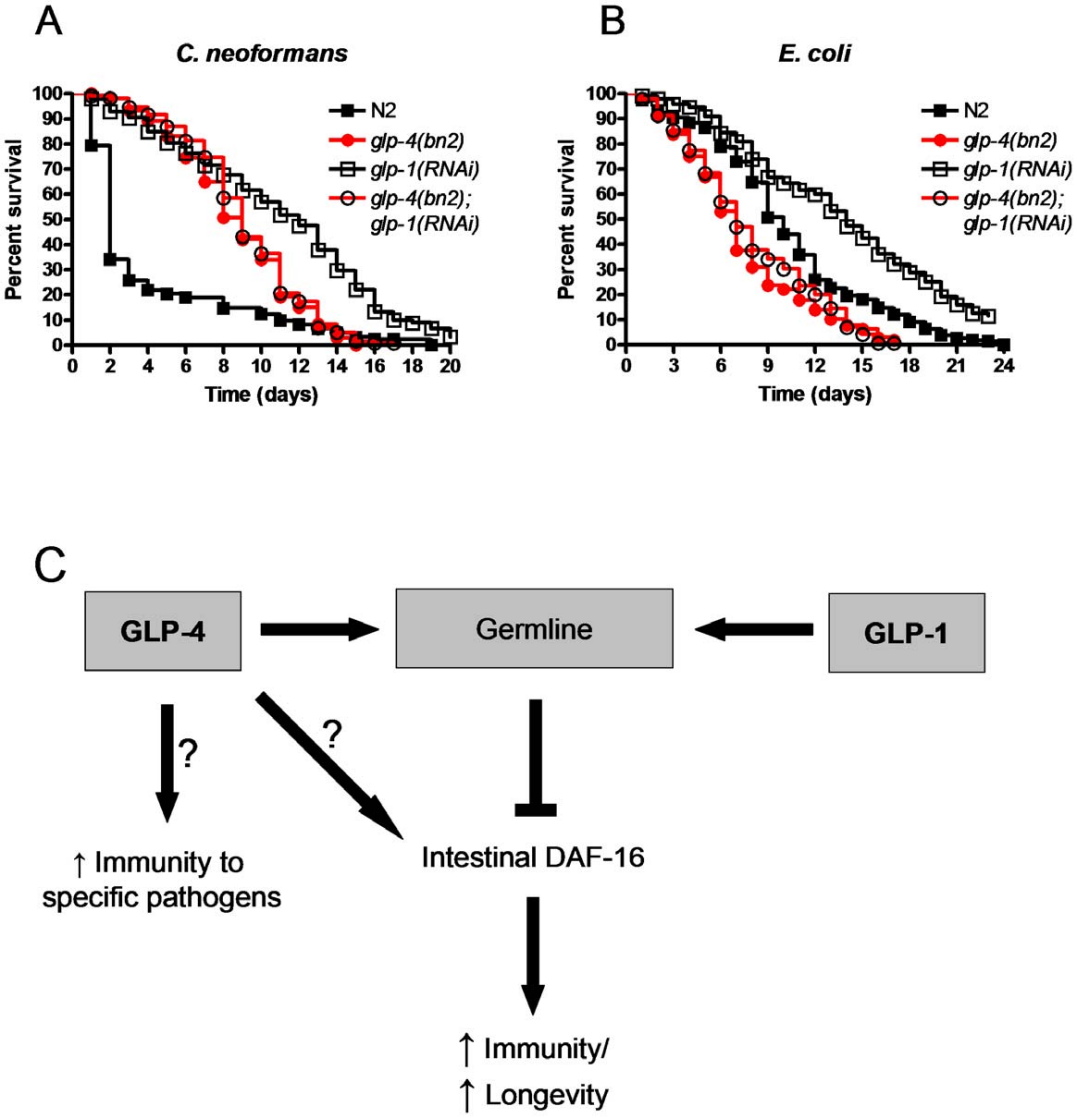
*glp-1* RNAi enhances longevity and pathogen resistance in wild-type animals but not in *glp-4* animals. Wild-type and *glp-4(bn2)* mutant nematodes were grown on *E. coli* carrying a vector control plasmid or expressing *glp-1* dsRNA were exposed to (**A**) *C. neoformans* or (**B**) *E. coli*. When compared to wild-type, *glp-1(RNAi)* showed significant differences on both *C. neoformans* (P<0.0001) and *E. coli* (P<0.0001). No significant differences were observed when *glp-4(bn2)* mutants were compared to *glp-4(bn2);glp-1(RNAi)* animals on either *C. neoformans* (P = 0.2257) or *E. coli* (P = 0.2525). 240–420 nematodes were used for each condition. (**C**) A proposed model indicating the role of *glp-4* in regulating *C. elegans* immunity. In this model, knocking out either *glp-1* or *glp-4* function leads to failure of the germline to develop. This in turn removes the inhibition of intestinal DAF-16, leading to increased immunity and longevity. However, the *glp-4* mutant has either a separate role in modulating immunity to specific pathogens which is also lost when *glp-4* is knocked out or is important in the activation of intestinal DAF-16 leading to less DAF-16 activation and a reduction in immune function.

Decreasing *glp-1* gene expression via RNAi in N2 wild-type nematodes enhances resistance to *C. neoformans* when compared to N2 wild-type animals treated with vector control (Figure 5A). However, *glp-1 RNAi* does not appear to have an effect on the resistance to *C. neoformans* in a *glp-4* mutant background (Figure 5A). In addition, *glp-1* RNAi significantly enhances survival in N2 wild-type animals but not in *glp-4* mutants (Figure 5B). These assays were also performed by treating *glp-1* nematodes with *glp-1* RNAi to serve as a control for any extraneous effects of the RNAi treatment; we observed no difference in survival on either *E. coli* or *C. neoformans* between *glp-1* mutants treated with *glp-1* RNAi or those treated with vector control (data not shown). As shown in Figures 3A, 3B, and 3C, *glp-4* animals are sensitive to *daf-16* RNAi. In addition, RNAi-mediated inhibition of ELT-2, which is a key transcription factor required for immunity against different microorganism including *Cryptococcus neoformans* [15], suppresses the enhanced resistance to *C. neoformans* of *glp-4* animals (Figure S1).

Taken together, our results indicate that both *glp-1* and *glp-4* mutations exhibit higher DAF-16 activity due to the lack of inhibition by the germline and that immunity against certain microorganisms may be affected in *glp-4* mutants by germline-independent mechanisms (Figure 5C). Both, *glp-1* and *glp-4* mutants grown at the restrictive temperature are severely depleted in germ cells as they do not contain more than approximately 15 germ nuclei, in contrast to the ∼1000 present in the single arm of the adult germline of wild-type animals. In *glp-1* mutants, germ cells that would normally remain in mitosis and continue to divide, enter meiosis leaving the animals with 5 to 15 germ cells that resemble sperm cells [22], [28]. The *glp-4* mutants have an average of 12 germ cells, which appear to be arrested at prophase during the mitotic cycle [29]. Thus, it seems unlikely that subtle variations in the germline between the *glp-1* and *glp-4* mutants may account for differences in resistance to certain microorganisms. Like *glp-1* mutants, germline-deficient *mes-1* mutants also exhibit an increased lifespan when grown on live *E. coli* [8], [19], indicating that the absence of the germline increases longevity. The reduced resistance to *E. coli* and *S. enterica* of *glp-4* mutants suggests that the mutation may affect a germline-independent mechanism involved in the regulation of innate immunity.

## Discussion

The development of the germline in *C. elegans* extracts a cost in terms of the longevity and immune function; previous work had demonstrated this cost in terms of increased resistance to *P. aeruginosa* and *S. marcescens* for animals lacking a germline [8], [9], [19], [20]. Our results indicate that the increased immunity of the germline-deficient mutants is indiscriminate as the animals without germlines have an enhanced resistance to a wide range of pathogens including Gram positive bacteria, Gram negative bacteria, and fungi.

It remains unclear exactly how interplay between the reproductive system and immune function occurs in wild-type nematodes with a germline. A new study, though, has demonstrated differing levels of expression of the gene *gld-1* when exposed to different species of bacteria expressed in grassland soil [30]. GLD-1 functions to limit proliferation of germ cells [31], [32], and as such, it is situated to help integrate signals from the nematode, including immune responses, and control reproduction [30]. Additionally, since targets of DAF-16 have been shown to suppress *gld-1*-induced tumors [33], [34], it seems likely that immune responses may help influence reproduction [30]. Further support for the interaction of immunity and reproduction can be seen in response to *S. enterica* infection, which induces the programmed cell death pathway and germ cell apoptosis [35]. These results, combined with results demonstrating that the nervous system plays a key role in modulating *C. elegans* immunity [36], [37], [38], [39], suggest that *C. elegans* utilizes multiple organ systems to form an integrated response to pathogens across the whole organism.

The enhanced longevity and pathogen-resistance of *daf-2* or germline-deficient mutants require DAF-16 [8], [9], [10], [11], [12], [14], [16]. However, DAF-16 inhibition by mutation or RNAi in a wild-type background does not affect the susceptibility of the nematodes to numerous pathogens, including *P. aeruginosa* [19], [20], *E. faecalis* [14], and *C. neoformans* [15]. A recent study indicates that DAF-16 plays a role in maintaining a basal level of immunity, but that it does not appear to be induced by pathogens; instead other immune signaling pathways appear to be induced by pathogen-exposure [40]. Thus, a potential explanation for the shortened lifespan of *daf-16(RNAi)* nematodes on both live and heat-killed *E. coli* but not on other microorganisms is that the presence of certain microorganisms activates other signaling pathways that help extend the survival lifespan of the nematode.

Previous studies have suggested that increased activation of DAF-16 was critical for the enhanced resistance to *P. aeruginosa* of germline-deficient animals [20], [21]. However, it has recently been suggested that DAF-16 may not be entirely responsible for the enhanced immunity observed in germline-deficient animals [19]. In this study, the resistance to *P. aeruginosa-*mediated killing of *glp-1* mutants was comparable to that of *glp-1;daf-16* double mutants, when the bacterial lawns were grown at room temperature instead of at 37°C [19]. It remains to be studied why *daf-16* mutations suppress the enhanced resistance to *P. aeruginosa-*mediated killing of *glp-1* only when bacterial lawns are grown under certain conditions [19].

It appears that DAF-16 plays a key role in defense response against *C. neoformans* in germline-deficient mutants. In wild-type nematodes, about one third of the animals exhibited DAF-16 activation after 24 hour exposure to *E. coli* as adults. This figure drops by approximately 10-fold if the animals are exposed to *C. neoformans* for 24 hours instead of *E. coli* (Figure 4C). This drop in DAF-16 activation is not observed in the two germline-deficient nematodes we tested, the *glp-1* and *glp-4* mutants. It is unclear how or why the drop in DAF-16 activation occurs in wild-type nematodes. One possibility is that *C. neoformans* is able to down-regulate DAF-16 expression in wild-type nematodes, but that this process is avoided in the germline-deficient nematodes. Further studies will be required to understand the mechanisms by which the *glp-4* mutation may affect DAF-16-dependent and –independent immune responses against pathogen infection.

## Materials and Methods

### Microbial and Nematode Strains

The following strains were used: *Escherichia coli* OP50 [41], *Salmonella enterica* serovar *typhimurium* SL1344 [42], *Enterococcus faecalis* OG1RF [43], *Cryptococcus neoformans* H99 [44], and *Pseudomonas aeruginosa* PA14 [45]. *C. elegans* strains utilized were wild-type N2, *glp-1(e2141)*, *glp-4(bn2)*, *fer-1(hc1)*, *fer-15(hc15)*. These strains were originally obtained from the Caenorhabditis Genetics Center and were maintained as hermaphrodites at 15°C, grown on modified NG agar plates and fed with *E. coli* strain OP50 as described [41].

### Transgenic Animals

The *Pgly-19:daf-16:gfp* transgenic animal was obtained from the Wolkow laboratory [27]. A *gcy-7:gfp* transgene, which is expressed in one or two head neurons, was also present in this strain as a co-injection marker (Wolkow, CA personal communication). This strain was then backcrossed to our laboratory's strain of wild-type N2 animals three times to standardize the genetic background. The *glp-1* and *glp-4* strains were generated by crossing the backcrossed transgenic animal *Pgly19::daf-16::gfp* to the *glp-1* and *glp-4* strains in our laboratory. Results of individual experiments can be found in Table S1.

### *C. elegans* killing assays

Cultures for the killing assays were grown in Luria-Bertani (LB) broth, except for *C. neoformans* H99 and *E. faecalis* OG1RF which were grown in yeast peptone dextrose (YPD) and brain-heart infusion (BHI) broth, respectively. All pathogens were grown at 37°C except *C. neoformans,* which was grown at 30°C. The pathogen lawns for the *C. elegans* killing assays were prepared by spreading 10–20 µl of an overnight culture of the bacterial strains on modified NG agar medium (0.35% peptone) in 3.5 cm or 6 cm diameter Petri plates. *C. neoformans* and *E. faecalis* were plated on BHI with 50 µg/ml gentamycin. Plates were incubated overnight before seeding them with young adult animals. These young adult animals were generated by placing gravid adults on NGM plates with lawns of *E. coli* OP50 and letting them lay eggs at 15°C for 6–10 hours. The gravid adults were then removed, and the eggs on the plate were allowed to develop at 25°C for 2.5 days to produce the young adults. The killing assays were performed at 25°C and animals were transferred once a day to fresh plates, until no more progeny were evident. Additional transfers were done after that point as needed to replenish food sources and to prevent the plates from drying out. The germline-deficient mutants were transferred at the same time as the N2 wild-type to maintain consistency. Animals were scored at the times indicated and were considered dead upon failure to respond to touch.

### *C. elegans* aging assays

*E. coli* OP50 was cultured in 50 ml of LB broth overnight at 37°C. The bacteria were then spun down and resuspended in 5 ml of LB broth. The *E. coli* were then heat-killed by placing the resuspended culture at 70°C for 3 hours. Twenty µl of this culture of heat-killed bacteria were then spread on modified NGM plates containing 0.35% peptone, 100 µg/ml 5-fluorodeoxyuridine (FUdR) [46], and 50 µg/ml gentamycin and allowed to incubate at 37°C overnight. Young adult animals were seeded onto these plates, and scored as indicated previously.

### RNA interference

RNA interference was used to generate loss-of-function RNAi phenotypes by feeding nematodes with *E. coli* strain HT115(DE3) expressing dsRNA that is homologous to a target gene [47], [48]. Briefly, *E. coli* with the appropriate vectors were grown in LB broth containing ampicillin (100 µg/ml) at 37°C overnight. RNAi plates were then generated by spreading these *E. coli* onto NGM plates containing 100 µg/ml ampicillin and 10 mM Isopropyl β-D-thiogalactoside (IPTG) to induce dsRNA expression, and the *E. coli* were allowed to grow on these plates overnight at 37°C.

L4 animals were placed on RNAi plates generated as described above and were allowed to develop into gravid adults at 15°C. Once these animals were gravid, they were transferred to fresh RNAi plates where they were allowed to lay eggs for 6–10 hours at 15°C. The gravid adults were then removed, and the eggs and plates were transferred to 25°C. The eggs were allowed to develop at 25°C for 2.5 days at which time they were seeded onto experimental plates and used as described above. *unc-22* RNAi was used as a positive control for the creation of loss-of-function phenotypes.

### Statistical analyses

Animal survival was plotted as a staircase curve using the PRISM (version 4.00) computer program. Survival curves are considered significantly different than the control when P values are less than 0.05. Prism uses the product limit or Kaplan-Meier method to calculate survival fractions and the logrank test, which is equivalent to the Mantel-Heanszel test, to compare survival curves.

### DAF-16 localization assays

Experimental plates featuring lawns of *E. coli* OP50 or *C. neoformans* H99 were generated as described in the *C. elegans* killing assays subsection. The transgenic animals expressing *Pgly-19::daf-16::gfp* were generated as described earlier, and young adults were generated as described in the *C. elegans* killing assays subsection. These young adult transgenic animals were then transferred to the experimental plates and left at 25°C for 24 hours. The animals were then visualized using a Leica MZ FLIII fluorescence stereomicroscope where they were categorized as predominately nuclear if at least five distinct nuclei were observed (as seen in Figure 4A) or predominately cytoplasmic (as seen in Figure 4B) as described in Berman, *et al*. (2006). Eight to ten plates of nematodes were scored over multiple, independent days, and the percentage of nematodes that were predominately nuclear was determined for each plate. Means and standard deviations were then calculated for each condition. Different conditions were compared using a two-tailed Mann-Whitney test (calculated by PRISM software) with a p<0.05 being considered significant.

## Supporting Information

Figure S1 *glp-4* mutant nematodes respond to *elt-2* RNAi. Wild-type and *glp-4(bn2)* mutant nematodes grown on *E. coli* carrying a vector control plasmid or expressing *elt-2* dsRNA were exposed to (A) *C. neoformans* or (B) *E. coli.* Significant differences were found when *glp-4(bn2);elt-2(RNAi)* worms were compared to vector control-treated *glp-4(bn2)* nematodes on *C. neoformans* (P<0.0001) and *E. coli* (P = 0.0004). 20–120 nematodes were used for each condition.
(0.60 MB TIF)

Table S1 Individual trial data from killing assays.
(0.03 MB XLS)

## References

[1] Davies S, Kattel R, Bhatia B, Petherwick A, Chapman T (2005). The effect of diet, sex and mating status on longevity in Mediterranean fruit flies (*Ceratitis capitata*), Diptera: Tephritidae.. Exp Gerontol.

[2] Westendorp RG, Kirkwood TB (1998). Human longevity at the cost of reproductive success.. Nature.

[3] Fedorka KM, Linder JE, Winterhalter W, Promislow D (2007). Post-mating disparity between potential and realized immune response in *Drosophila melanogaster*.. Proc Biol Sci.

[4] Fedorka KM, Zuk M, Mousseau TA (2004). Immune suppression and the cost of reproduction in the ground cricket, *Allonemobius socius*.. Evolution.

[5] Gwynn DM, Callaghan A, Gorham J, Walters KF, Fellowes MD (2005). Resistance is costly: trade-offs between immunity, fecundity and survival in the pea aphid.. Proc Biol Sci.

[6] McKean KA, Nunney L (2001). Increased sexual activity reduces male immune function in *Drosophila melanogaster*.. Proc Natl Acad Sci U S A.

[7] Mukhopadhyay A, Tissenbaum HA (2007). Reproduction and longevity: secrets revealed by *C. elegans*.. Trends Cell Biol.

[8] Arantes-Oliveira N, Apfeld J, Dillin A, Kenyon C (2002). Regulation of life-span by germ-line stem cells in *Caenorhabditis elegans*.. Science.

[9] Hsin H, Kenyon C (1999). Signals from the reproductive system regulate the lifespan of *C. elegans*.. Nature.

[10] Libina N, Berman JR, Kenyon C (2003). Tissue-specific activities of *C. elegans* DAF-16 in the regulation of lifespan.. Cell.

[11] Lin K, Hsin H, Libina N, Kenyon C (2001). Regulation of the *Caenorhabditis elegans* longevity protein DAF-16 by insulin/IGF-1 and germline signaling.. Nat Genet.

[12] Kenyon C, Chang J, Gensch E, Rudner A, Tabtiang R (1993). A *C. elegans* mutant that lives twice as long as wild type.. Nature.

[13] Anyanful A, Dolan-Livengood JM, Lewis T, Sheth S, Dezalia MN (2005). Paralysis and killing of *Caenorhabditis elegans* by enteropathogenic *Escherichia coli* requires the bacterial tryptophanase gene.. Mol Microbiol.

[14] Garsin DA, Villanueva JM, Begun J, Kim DH, Sifri CD (2003). Long-lived *C. elegans daf-2* mutants are resistant to bacterial pathogens.. Science.

[15] Kerry S, TeKippe M, Gaddis NC, Aballay A (2006). GATA transcription factor required for immunity to bacterial and fungal pathogens.. PLoS ONE.

[16] Singh V, Aballay A (2006). Heat-shock transcription factor (HSF)-1 pathway required for *Caenorhabditis elegans* immunity.. Proc Natl Acad Sci U S A.

[17] Singh V, Aballay A (2009). Regulation of DAF-16-mediated Innate Immunity in *Caenorhabditis elegans*.. J Biol Chem.

[18] Murphy CT, McCarroll SA, Bargmann CI, Fraser A, Kamath RS (2003). Genes that act downstream of DAF-16 to influence the lifespan of *Caenorhabditis elegans*.. Nature.

[19] Alper S, McElwee MK, Apfeld J, Lackford B, Freedman JH (2010). The *Caenorhabditis elegans* germ line regulates distinct signaling pathways to control lifespan and innate immunity.. J Biol Chem.

[20] Miyata S, Begun J, Troemel ER, Ausubel FM (2008). DAF-16-dependent suppression of immunity during reproduction in *Caenorhabditis elegans*.. Genetics.

[21] Evans EA, Kawli T, Tan MW (2008). *Pseudomonas aeruginosa* suppresses host immunity by activating the DAF-2 insulin-like signaling pathway in *Caenorhabditis elegans*.. PLoS Pathog.

[22] Austin J, Kimble J (1987). *glp-1* is required in the germ line for regulation of the decision between mitosis and meiosis in *C. elegans*.. Cell.

[23] Crittenden SL, Troemel ER, Evans TC, Kimble J (1994). GLP-1 is localized to the mitotic region of the *C. elegans* germ line.. Development.

[24] Roberts TM, Ward S (1982). Membrane flow during nematode spermiogenesis.. J Cell Biol.

[25] Ward S, Miwa J (1978). Characterization of temperature-sensitive, fertilization-defective mutants of the nematode *Cenorhabditis elegans*.. Genetics.

[26] Berman JR, Kenyon C (2006). Germ-cell loss extends *C. elegans* life span through regulation of DAF-16 by kri-1 and lipophilic-hormone signaling.. Cell.

[27] Gami MS, Iser WB, Hanselman KB, Wolkow CA (2006). Activated AKT/PKB signaling in *C. elegans* uncouples temporally distinct outputs of DAF-2/insulin-like signaling.. BMC Dev Biol.

[28] Priess JR, Schnabel H, Schnabel R (1987). The *glp-1* locus and cellular interactions in early *C. elegans* embryos.. Cell.

[29] Beanan MJ, Strome S (1992). Characterization of a germ-line proliferation mutation in *C. elegans*.. Development.

[30] Coolon JD, Jones KL, Todd TC, Carr BC, Herman MA (2009). *Caenorhabditis elegans* genomic response to soil bacteria predicts environment-specific genetic effects on life history traits.. PLoS Genet.

[31] Francis R, Barton MK, Kimble J, Schedl T (1995). *gld-1*, a tumor suppressor gene required for oocyte development in *Caenorhabditis elegans*.. Genetics.

[32] Francis R, Maine E, Schedl T (1995). Analysis of the multiple roles of *gld-1* in germline development: interactions with the sex determination cascade and the *glp-1* signaling pathway.. Genetics.

[33] Pinkston JM, Garigan D, Hansen M, Kenyon C (2006). Mutations that increase the life span of *C. elegans* inhibit tumor growth.. Science.

[34] Pinkston-Gosse J, Kenyon C (2007). DAF-16/FOXO targets genes that regulate tumor growth in *Caenorhabditis elegans*.. Nat Genet.

[35] Aballay A, Ausubel FM (2001). Programmed cell death mediated by *ced-3* and *ced-4* protects *Caenorhabditis elegans* from *Salmonella typhimurium*-mediated killing.. Proc Natl Acad Sci U S A.

[36] Anyanful A, Easley KA, Benian GM, Kalman D (2009). Conditioning protects *C. elegans* from lethal effects of enteropathogenic *E. coli* by activating genes that regulate lifespan and innate immunity.. Cell Host Microbe.

[37] Kawli T, Tan MW (2008). Neuroendocrine signals modulate the innate immunity of *Caenorhabditis elegans* through insulin signaling.. Nat Immunol.

[38] Styer KL, Singh V, Macosko E, Steele SE, Bargmann CI (2008). Innate immunity in *Caenorhabditis elegans* is regulated by neurons expressing NPR-1/GPCR.. Science.

[39] Zugasti O, Ewbank JJ (2009). Neuroimmune regulation of antimicrobial peptide expression by a noncanonical TGF-beta signaling pathway in *Caenorhabditis elegans* epidermis.. Nat Immunol.

[40] Shivers RP, Youngman MJ, Kim DH (2008). Transcriptional responses to pathogens in *Caenorhabditis elegans*.. Curr Opin Microbiol.

[41] Brenner S (1974). The genetics of *Caenorhabditis elegans*.. Genetics.

[42] Wray C, Sojka WJ (1978). Experimental *Salmonella typhimurium* infection in calves.. Res Vet Sci.

[43] Murray BE, Singh KV, Ross RP, Heath JD, Dunny GM (1993). Generation of restriction map of *Enterococcus faecalis* OG1 and investigation of growth requirements and regions encoding biosynthetic function.. J Bacteriol.

[44] Franzot SP, Salkin IF, Casadevall A (1999). *Cryptococcus neoformans* var. grubii: separate varietal status for *Cryptococcus neoformans* serotype A isolates.. J Clin Microbiol.

[45] Tan MW, Mahajan-Miklos S, Ausubel FM (1999). Killing of *Caenorhabditis elegans* by *Pseudomonas aeruginosa* used to model mammalian bacterial pathogenesis.. Proc Natl Acad Sci U S A.

[46] Gandhi S, Santelli J, Mitchell DH, Stiles JW, Sanadi DR (1980). A simple method for maintaining large, aging populations of *Caenorhabditis elegans*.. Mech Ageing Dev.

[47] Fraser AG, Kamath RS, Zipperlen P, Martinez-Campos M, Sohrmann M (2000). Functional genomic analysis of *C. elegans* chromosome I by systematic RNA interference.. Nature.

[48] Timmons L, Fire A (1998). Specific interference by ingested dsRNA.. Nature.

