# Supplementary figures and images for: C. elegans Germline-Deficient Mutants Respond to Pathogen Infection Using Shared and Distinct Mechanisms
Source: PLoS One. 2010 Jul 26;5(7):e11777. doi: 10.1371/journal.pone.0011777 (PMC2909909; doi:10.1371/journal.pone.0011777)

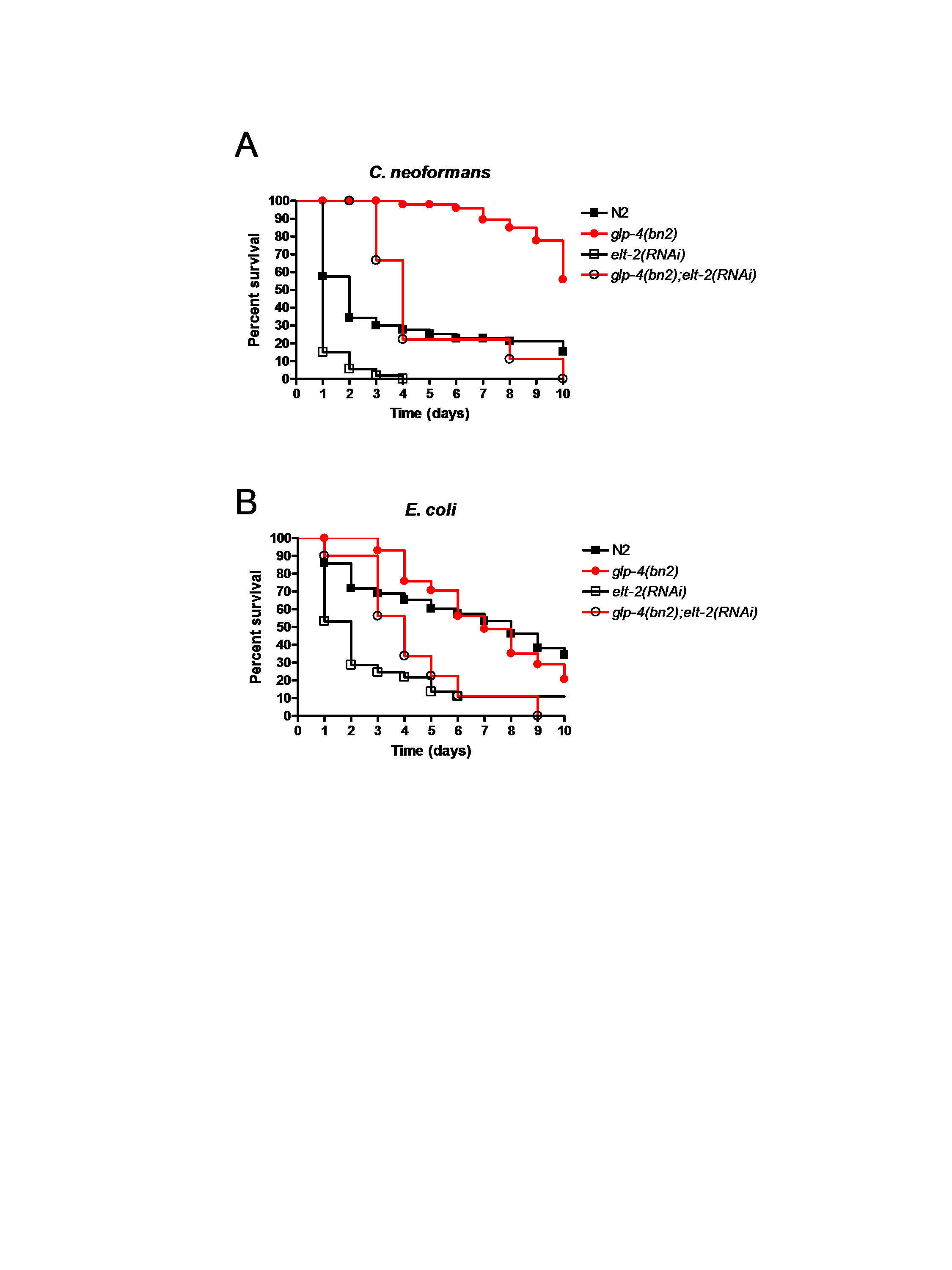

Supplement: Figure S1 — glp-4 mutant nematodes respond to elt-2 RNAi. Wild-type and glp-4(bn2) mutant nematodes grown on E. coli carrying a vector control plasmid or expressing elt-2 dsRNA were exposed to (A) C. neoformans or (B) E. coli. Significant differences were found when glp-4(bn2);elt-2(RNAi) worms were compared to vector control-treated glp-4(bn2) nematodes on C. neoformans (P<0.0001) and E. coli (P = 0.0004). 20–120 nematodes were used for each condition. (0.60 MB TIF) [file pone.0011777.s001.tif]
